# A promiscuous glycosyltransferase generates poly-β-1,4-glucan derivatives that facilitate mass spectrometry-based detection of cellulolytic enzymes†

**DOI:** 10.1039/d1ob00971k

**Published:** 2021-06-01

**Authors:** Gregory S. Bulmer, Ashley P. Mattey, Fabio Parmeggiani, Ryan Williams, Helene Ledru, Andrea Marchesi, Lisa S. Seibt, Peter Both, Kun Huang, M. Carmen Galan, Sabine L. Flitsch, Anthony P. Green, Jolanda M. van Munster

**Affiliations:** Manchester Institute of Biotechnology (MIB) & School of Natural Sciences, The University of Manchester Manchester UK jolanda.vanmunster@manchester.ac.uk jolanda.van-munster@sruc.ac.uk; Department of Chemistry, Materials and Chemical Engineering “G. Natta”, Politecnico di Milano Milano Italy; School of Chemistry, University of Bristol Bristol UK; Scotland's Rural College, Central Faculty Edinburgh UK

## Abstract

Promiscuous activity of a glycosyltransferase was exploited to polymerise glucose from UDP-glucose *via* the generation of β-1,4-glycosidic linkages. The biocatalyst was incorporated into biocatalytic cascades and chemo-enzymatic strategies to synthesise cello-oligosaccharides with tailored functionalities on a scale suitable for employment in mass spectrometry-based assays. The resulting glycan structures enabled reporting of the activity and selectivity of celluloltic enzymes.

## Abstract 2

Promiscuous activity of a glycosyltransferase was exploited to polymerise glucose from UDP-glucose *via* the generation of β-1,4-glycosidic linkages.

## Graphical abstract

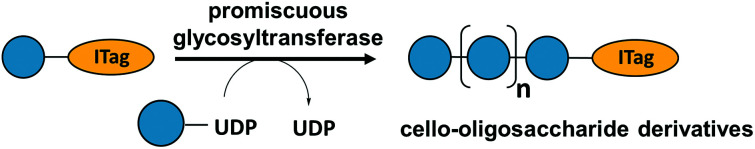

Cellulose, a linear polysaccharide consisting of β-1,4-linked glucose, is of critical importance in biotechnology, nutrition and microbial pathogenicity. As the major structural component of plant cell walls, it is exploited as a renewable resource in the bio-economy, enabling sustainable production of fuels and chemicals.^1^ Cellulose and its oligosaccharides play key roles in health and disease, for example as dietary fiber^2^ and have a broad range of applications as biosurfactants, nanomaterials and biogels.^3^ The production of cellulose-based structures has therefore attracted much attention.

Chemical synthesis of cellulose derivatives is complex due to the required stereo- and regioselectivity.^4^ Enzymatic synthesis of cellulose *in vitro* is challenging because the natural biosynthetic machinery consists of membrane-embedded, multi component systems.^5^ Native and derivatised cellulose (oligosaccharides) have been generated from glucose-1-phosphate and cellobiose *via* the reversible reaction mechanism of cellodextrin phosphorylases, whereby reaction conditions can direct the degree of polymerisation (DP).^6–8^ Furthermore, exploitation of enzymes that utilise activated glycosyl donors (*e.g.* glycosyl fluorides) such as glycosynthases, have opened up a raft of new options for synthesising glycosides.^9–11^ However, such systems require unnatural donors or are biased towards production of very long oligomers. Despite these advances, it remains challenging to generate soluble cello-oligosaccharides from natural donors due to the limited availability of suitable biocatalysts.

Glycosyltransferases (GT) synthesise highly regio- and stereospecific glycosidic bonds between glycan acceptors and activated sugar donors.^12^ Enzymatic synthesis of Glc-β-1,4-Glc linkages from nucleotide sugars has been restricted to plant and bacterial cellulose synthases. However, GTs can be promiscuous in acceptor and donor substrates, thereby providing biocatalysts with potentially exploitable side reactions with rates that in many cases were demonstrated to be sufficient for exploitation in glycoside synthesis.^13–15^ Therefore, an GT able to accommodate both UDP-Glc as donor and a Glc-terminated structure as acceptor would in effect function as glucose polymerase (Fig. 1A), whereby the DP may be tuned *via* reaction optimisation or enzyme engineering.

**Fig. 1:**
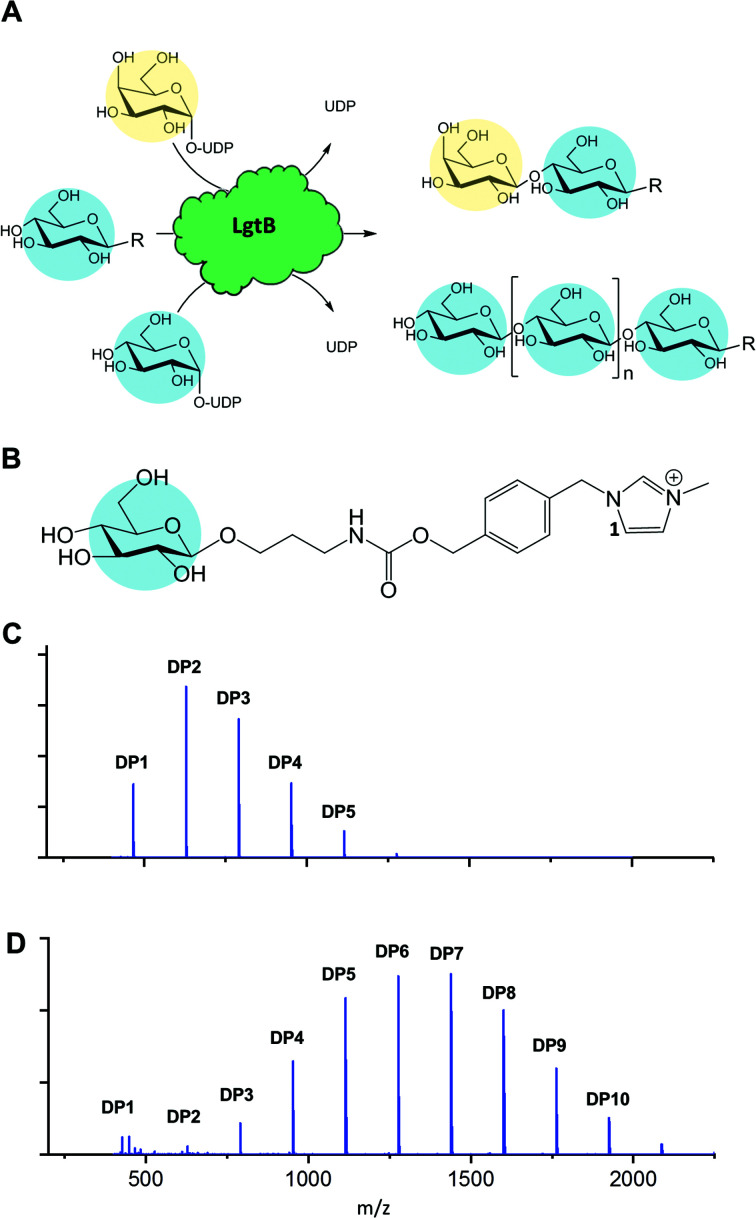
Promiscuous galactosyltransferase activity results in β-1,4-linked glucose polymerisation. A, Reactions catalysed by biocatalyst LgtB, blue and yellow circles represent Glc and Gal respectively, following symbol nomenclature for glycans.^28,29^ B, LgtB acceptor substrate for LgtB, glucoside derivatised with ITag-1 (**1**). C, Incubation length and UDP-Glc concentration alters length and ratio of oligosaccharides produced, shown after 2 d with Glc-ITag-1 (**1**), 1.5 mM UDP-Glc and D, after 7 d with 15 mM UDP-Glc.

Here we describe how the rational exploration of galactosyltransferase substrate promiscuity resulted in the identification of a broad-specificity biocatalyst that functions as a glucose polymerase *in vitro*. We demonstrate the synthesis of cello-oligosaccharides and their derivatives to a scale suitable for mass spectrometry-based detection and exemplify how these compounds facilitate the profiling of hydrolytic and oxidative biomass-degrading enzyme activities.

With the aim to identify a GT capable of generating β-1,4-linked glucose (Glc) oligosaccharides, we assembled a panel of five recombinantly expressed galactosyltransferases and screened it for promiscuous acceptance of Glc-based donor and acceptor substrates. Transfer of UDP-Glc to an acceptor with a terminal Glc-R motif would result in reaction products that can be re-used as an acceptor, thus enabling the desired Glc polymerisation. As the acceptor structure can potentially affect the generated glycosidic linkage type,^16^ enzymes with a variety of reported specificities were included.

To monitor enzyme activity we employed a sensitive and fast assay based on glycosylation of sugar acceptors labelled with imidazolium-based probes (ITags), such as 4-(1-methyl-3-methyleneimidazolium)benzyl carbamate β-glucoside^15,17^ Glc-ITag-1 (**1**) (Fig. 1B). Such cationic ITags generate strong signals in mass-spectrometry that dominate the analyte ionisation and can be used as soluble handles that support immobilisation and purification of glycosides during chemical derivatisation.^18,19^ To aid subsequent assessment of the type of glycosidic linkage produced during polymerisation, we chemically synthesised the less complex, novel 4-(1-methyl-3-methyleneimidazolium) benzyl β-glucoside Glc-ITag-2 (**2**) (Fig. S1A†) in 4 steps (Fig. S5†) with a yield of 49%. The transfer of galactose (Gal) from UDP-Gal to ITagged acceptors was detected as an activity displayed by all tested panel members (Fig. S1 and S2†). In contrast, activity with UDP-Glc was detected only for *Homo sapiens* B4GALT4 and *Neisseria meningitidis* LgtB, with multiple reaction products due to polymerisation observed in the latter case. LgtB was therefore identified as the biocatalyst most suitable for our target activity, *i.e.* glucose polymerisation.

After incubation of LgtB with **1** or **2** and an excess of UDP-Glc at 37 °C, a range of ITagged glucose oligosaccharides with DPs of up to 7 and 9, respectively (Fig. S9†) were observed. Adjustment of reaction conditions such as incubation time and donor concentration allowed direction of glucose polymerisation towards a desired product range (Fig. 1C, D and S6†). In reactions optimised towards high DP products, a ≥99% conversion of the initial acceptor (based on Maldi-ToF MS spectral intensity) into oligosaccharides of up to DP12 was detected. Chromatographic separation of the generated oligosaccharides proved challenging in our hands due to the chemical and structural similarities between the products and the limited amount of material available, and therefore the isolation and quantification of individual oligosaccharides and determination of their isolated yields could not be realised within the project constrains. 2D HSQC NMR analysis of purified ITagged oligosaccharides generated from (**2**) confirmed that the Glc residues were connected by β-1,4-glycosidic linkages (Fig. S8†), demonstrating the strict selectivity in anomeric configuration and position of the glycoside linkage that is formed by LgtB.

LgtB was also able to polymerise Glc onto a broad range of other acceptor substrates (Table S1†) including native cello-oligosaccharides and derivatives with reducing end conjugates *e.g.* Glc(n)-pNP (Fig. S4†). This agrees with the reported broad acceptor substrate scope of this enzyme, which has been exploited for chemo-enzymatic synthesis of β-1,4-linked galactosides incorporating *e.g.* GlcNAc(-pNP), Man-pNP, Glc(-pNP) and various C2-derivatives.^13–15,20^ No activity was found using Gal, xylose (Xyl), arabinose (Ara), lactose (Gal-β-1,4-Glc), trehalose (Glc-α-1,1-Glc) or UDP-Glc as acceptors. Acceptor substrates thus require an equatorial configuration of the C4 –OH group and the presence of a C6 –OH group while both the C1 and C2 substitutions are highly flexible. Limited transfer of Xyl and GalNAc but not GlcNAc from their respective UDP-conjugates to acceptors was also detected (Fig. S3†). Taken together, LgtB was identified as a promiscuous biocatalyst with glucose polymerase activity that can be exploited for polymerisation of glucose onto a broad range of acceptors on a scale suitable for detection by mass spectrometry.

Polymerisation allows cello-oligosaccharide biosynthesis in biocatalytic cascades, such as those enabling regeneration of UDP-glucose. We combined LgtB in a one pot, two enzyme cascade (Fig. S7†) with *Solanum lycopersicum* sucrose synthase (SLSUS6)^21^, hereafter SuSy. In the presence of UDP, SuSy converts relatively inexpensive sucrose into fructose and UDP-Glc, this biocatalyst is therefore widely employed for the synthesis of nucleotide sugars and glucosides.^22^ Incubation of SuSy with LgtB, sucrose and **1** or **2** resulted in formation of ITagged cello-oligosaccharides (Fig. S9†) with DP ranges and ratios similar to those observed using UDP-Glc directly. Thus, UDP-Glc generated *via* SuSy activity can be used as a donor substrate by LgtB, in a one-pot biocatalytic cascade that polymerises glucose from inexpensive sucrose.

To exemplify LgtB utility in mass-spectrometry based assays, we exploited ITagged glucose oligosaccharides generated *via* LgtB activity to rapidly profile activity of lytic polysaccharide monooxygenases (LPMOs), copper dependent enzymes that oxidatively degrade oligosaccharides and polysaccharides. LPMOs insert a single oxygen atom into the C1–H and/or C4–H bond of saccharide substrates, ultimately producing aldonic acids or 4-gemdiol-aldoses, respectively (Fig. S10†).^23^ This activity enables more cost-effective production of biofuels from lignocellulose^24^ and holds promise in carbohydrate functionalisation. Sensitive assays to profile libraries of LPMO variants with regard to activity on soluble oligosaccharides and selectivity for C1- *vs.* C4-oxidation, are therefore of interest. A small panel of LPMOs was produced in *E. coli* as the expression host; *Lentinus similis Ls*(AA9)A,^23^*Neurospora crassa Nc*LPMO9C^25^ and *Thermobifida fusca Tf*(AA10)B^26^ (see ESI† for detailed protocols). Biotransformations were performed with purified enzymes using an envelope of ITagged glucose oligosaccharides (DP 1–10) in the presence of H_2_O_2_ and ascorbate as a reducing agent. The distribution of labelled oligosaccharides was unchanged following incubation with *Tf*(AA10)B (Fig. 2A), confirming lack of activity towards the oxidation of soluble cello-oligosaccharides with DP < 10. Conversely, reactions with *Nc*(AA9)C and *Ls*(AA9)A led to complete consumption of oligosaccharides with DP ≥ 6 and 4, respectively (Fig. 2A), with concomitant formation of labelled C4-oxidised products (DP 2–4 and DP 2–3, respectively) with molecular weights of [M − 2] (C4-ketone) and [M + 16] (C4-gemdiol) (with M as the molecular weight of corresponding non-oxidised labelled carbohydrate) (Fig. 2B). These data are consistent with reported substrate profiles. Notably, the ITag cationic nature avoids formation of sodium or potassium adducts^15^ that complicate interpretation^30^ of MS-based detection of LPMO products. Selective detection of endo-cellulase activity in glycoside hydrolase (GH) preparations is in principle enabled *via* an enzyme substrate that cannot be hydrolysed from its termini. Exploiting LgtB, we generated such a ‘blocked’ substrate probe compatible with fast and sensitive detection of enzyme activity *via* MS. Initially we confirmed commercially available cellulolytic GHs degrade unmodified ITagged cello-oligosaccharides (Fig. 3A and B). ‘Blocked’ ITagged oligosaccharides were then generated *via* a chemo-enzymatic approach (see ESI† for detailed methods) based on the creation of a bio-orthogonal aldehyde on the non-reducing, Gal-capped termini of oligosaccharides. The aldehydes were subsequently derivatised with a nicotinic hydrazide group (Fig. S13B†) *via* previously described methodologies.^27^ Derivatisation of the reducing end, here accomplished *via* ITag, was required to ensure generation of a homogenous end product (Fig. S12†).

**Fig. 2:**
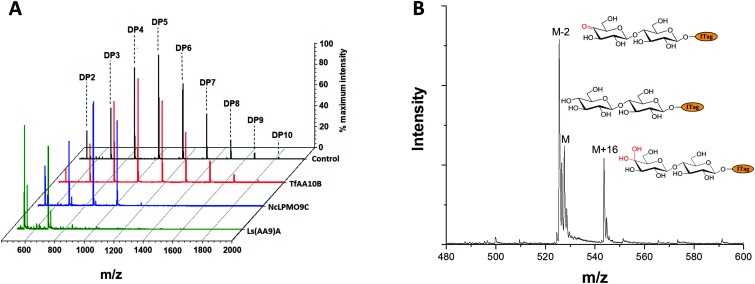
ITagged cello-oligosaccharides as probes for LPMO activity. A, Selective activity on ITagged glucose oligosaccharide substrates by a panel of LPMOs. B, The products of *Nc*LPMO9C activity on ITagged cello-oligosaccharides include those with a mass corresponding to C4-ketone (M − 2) and gemdiol (M + 16) disaccharides, confirming C4-oxidative activity.

**Fig. 3:**
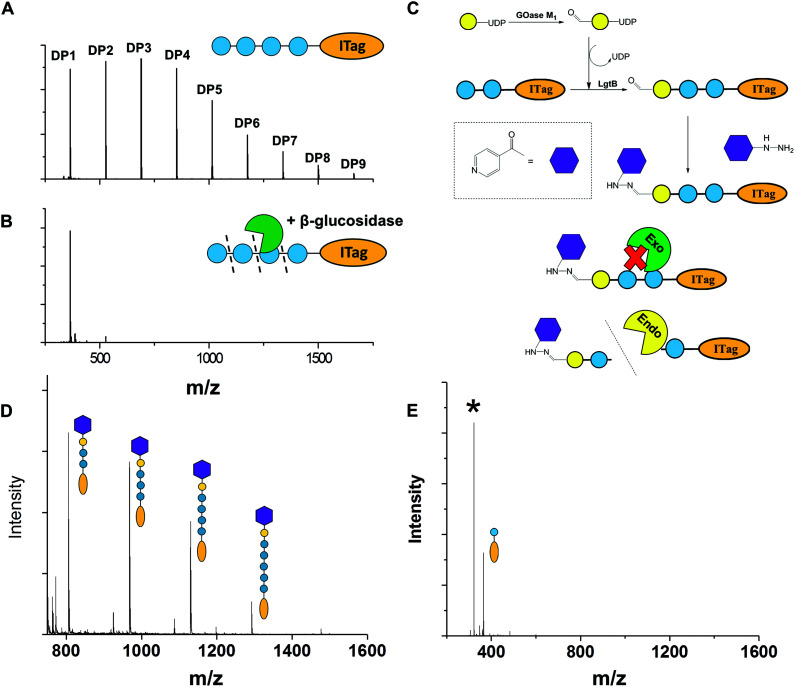
Chemo-enzymatic derivatisation of cello-oligosaccharides to selective endo-cellulase substrates. A, Representative MALDI-TOF MS spectra demonstrating ITagged cello-oligosaccharides after incubation without enzymes and B, with β-glucosidase from almond. C, Derivatisation strategy employed to protect LgtB product termini against hydrolytic activity; D, probes after oxidised galactose and nicotinic hydrazide ligation after incubation with β-glucosidase and β-galactosidase; E, same probes after incubation with *A. niger* cellulases. Asterix indicates a glycosylated hydrazide product.

With the blocked substrates in hand, we confirmed their protection against exo-acting enzyme activity. Terminal galactose addition was sufficient to protect cello-oligosaccharides against β-glucosidase activity, while hydrazide derivatisation was required and sufficient to protect structures against a set of β-galactosidases (Fig. 3D and Fig. S14†). Finally, we employed the blocked substrates to monitor endo-acting cellulases activity, and validated the presence of this activity by MALDI-TOF MS (Fig. 3E). The observed degradation profile was similar to that generated by the same enzyme incubated with unblocked oligosaccharides. We conclude these probes enable selective detection of endo-cellulase activity *via* MS.

## Conclusions

In conclusion, LgtB has a broad substrate scope that includes polymerase activity with UDP-Glc as donor and Glc-terminated glycosides as acceptors. We successfully exploited this promiscuous activity to polymerise glucose *via* β-1,4-glycosidic linkages onto a variety of acceptors containing chemical handles, producing functionalised, tailored cello-oligosaccharide derivatives as detectible by mass spectrometry. We demonstrated the incorporation of LgtB-mediated glucose polymerisation into chemo-enzymatic derivatisation strategies, *via* production of probes to selectively detect endo-acting cellulolytic enzyme activities in a sensitive, MS-based assay. Further work regarding scalability of the LgtB reaction including determination of isolated yields should confirm if LgtB would be suitable as catalyst for glucose polymerisation in reactions at preparative scale and upwards. As LgtB can be obtained *via* facile heterologous expression in *E. coli*, this biocatalyst for the synthesis of β-1,4-glycosidic linkages is very well suited to be engineered to accept desired non-natural substrates, which may open up novel routes to enzymatic synthesis of cellulose derivatives.

## Conflicts of interest

There are no conflicts to declare.

## Supplementary Material

OB-019-D1OB00971K-s001
