# Supplementary material for: A promiscuous glycosyltransferase generates poly-β-1,4-glucan derivatives that facilitate mass spectrometry-based detection of cellulolytic enzymes
Source: Org Biomol Chem. 2021 Jun 1;19(25):5529–33. doi: 10.1039/d1ob00971k (PMC8243248; doi:10.1039/d1ob00971k)
Supplement: OB-019-D1OB00971K-s001 [file OB-019-D1OB00971K-s001.pdf]

Electronic Supplementary Material (ESI) for Organic & Biomolecular Chemistry.
This journal is © The Royal Society of Chemistry 2021

Supporting information

A promiscuous glycosyltransferase generates poly-β-1,4-glucan derivatives that facilitate mass spectrometry-based detection of cellulolytic enzymes

Gregory S. Bulmer,[a] Ashley P. Mattey,[a] Fabio Parmeggiani,[a,c] Ryan Williams,[b] Helene Ledru,[b] Andrea Marchesi,[a] Lisa S. Seibt,[a] Peter Both,[a] Kun Huang,[a] M. Carmen Galan,[b] Sabine L. Flitsch,[a] Anthony P. Green[a] and Jolanda M. van Munster*[a,d]

[a] Manchester Institute of Biotechnology (MIB) & School of Natural Sciences, The University of Manchester, 131 Princess Street, Manchester, M1 7DN, United Kingdom

[b] School of Chemistry, University of Bristol, Cantock's Close, Bristol, BS8 ITS, United Kingdom

[c] Department of Chemistry, Materials and Chemical Engineering "G. Natta", Politecnico di Milano, Via Mancinelli 7, 20131, Milano, Italy.

[d] Scotland's Rural College, West Mains Road, Kings Buildings, Edinburgh, EH9 3JG, United Kingdom

Contents

Supplementary figure 1 | ITagged oligosaccharides and SNFG nomenclature used in this study. 3

Supplementary figure 2| Galactosylation of ITagged glycosides during activity screening. 4

Supplementary figure 3 | Donor scope assessment of LgtB with ITagged acceptors 6

Supplementary figure 4 | Glucose polymerisation occurs onto a range of acceptors 7

Supplementary figure 5 | Synthesis of 4-(1-Methyl-3-methyleneimidazolium)benzyl β-D-glucopyranoside trifluoromethanesulfonate (2) 8

Supplementary figure 6 | Modification of reaction conditions drives oligosaccharide profile distribution ..9

Supplementary figure 7 | Biocatalytic cascade enabling effective coupling of UDP-glucose regeneration with glucose polymerisation. 10

Supplementary figure 8 | HSQC confirmed that ITagged oligosaccharides generated by LgtB consist of β1,4-linked glucose 11

Supplementary figure 9 | SuSy cascade enables recycling of UDP for donor production 11

Supplementary figure 10 | LPMO reaction products 12

Supplementary figure 11 | Production of oxidised UDP-Gal 12

Supplementary figure 13| Galactosylation and hydrazide ligation of ITagged cello-oligosaccharides 13

Supplementary figure 14| Confirmation of β-galactosidase activity blocking 14

Materials and methods 15

Galactosyltransferase production 15

NMR of carbohydrates 16

Mass spectrometry of carbohydrates 16

SuSy system for UDP recycling 16

GalT screening panel 16

Donor scope assessment and ITagged oligosaccharide production using LgtB 17

Enzymatic digestions of ITagged-oligosaccharides 17

LPMO production 17

Endo-substrate development 18

Synthesis of 4-(1-Methyl-3-methyleneimidazolium)benzyl β-D-glucopyranoside trifluoromethanesulfonate (2) (Glc-ITag-2) 19

References 21

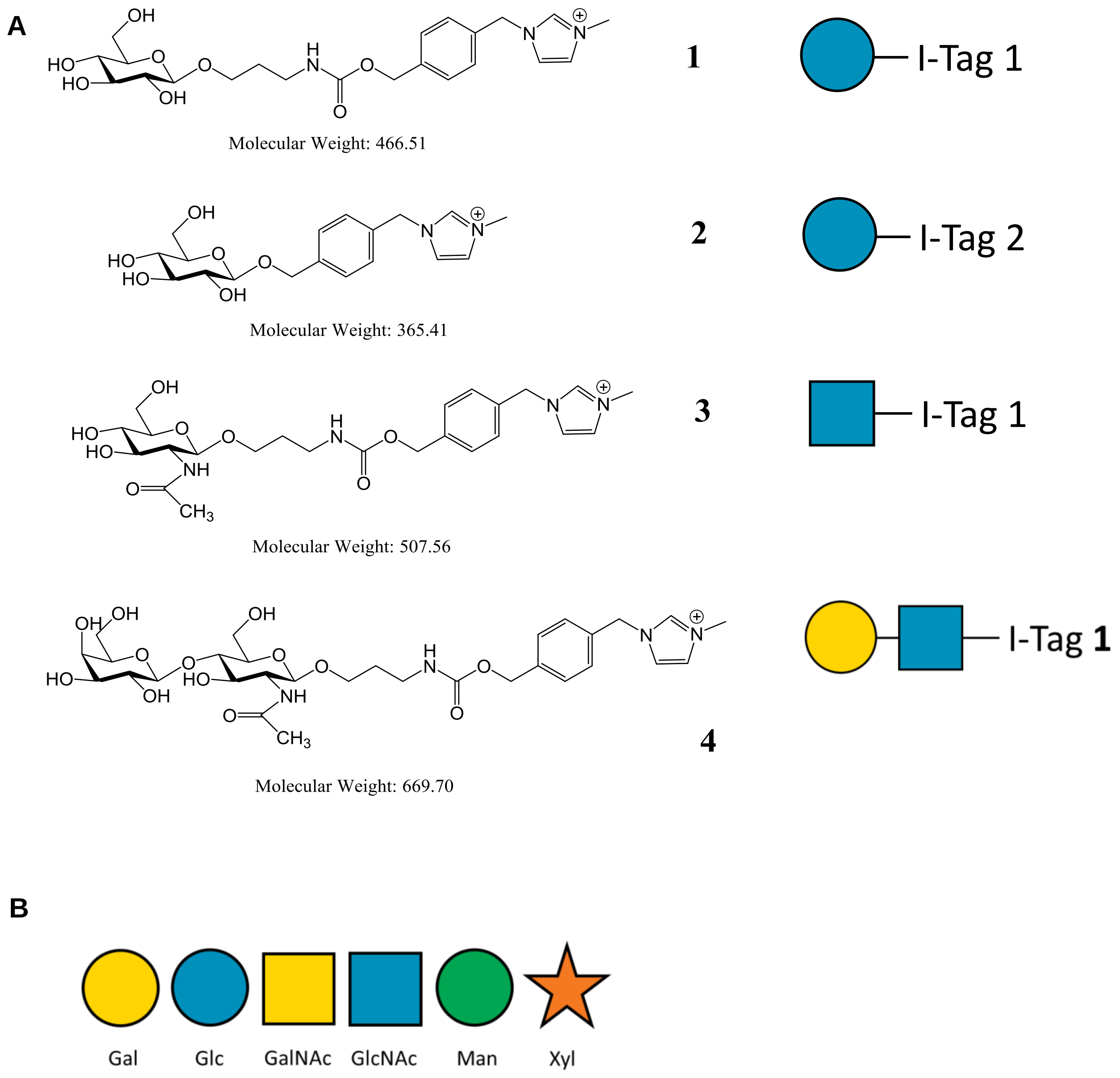

Supplementary figure 1 | ITagged oligosaccharides and SNFG nomenclature used in this study. **A**, Above compounds are referred to as follows: Glc-ITag-1 (**1**), Glc-ITag-2 (**2**), GlcNAc-ITag-1 (**3**) and LacNAc-ITag-1 (**4**). Compounds **1**, **3** and **4** were synthesised as previously described[1] **B**, Monosaccharides as defined by the SNFG with accepted abbreviation.

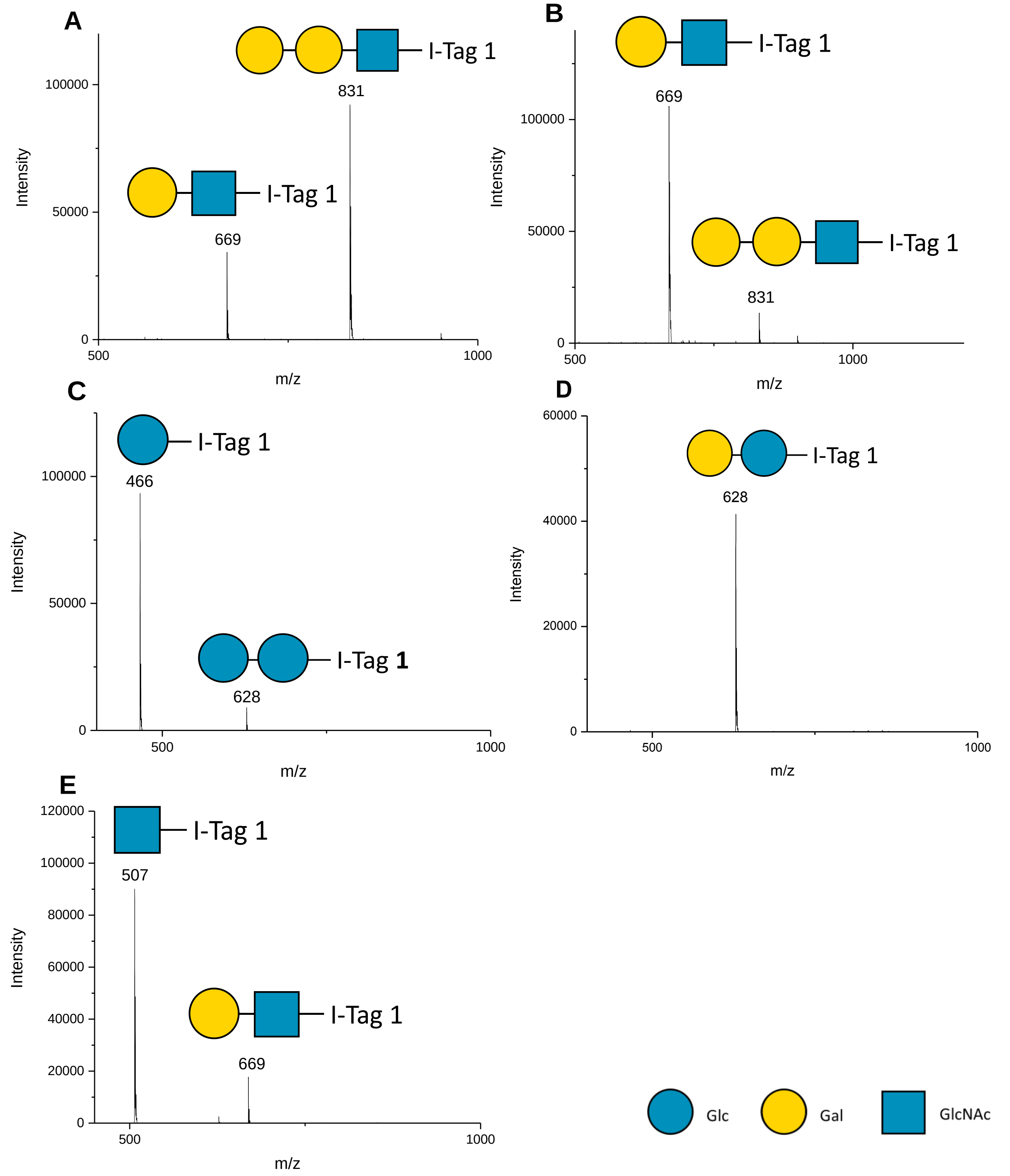

Supplementary figure 2| Galactosylation of ITagged glycosides during activity screening. Galactosylation activity of LgtC (**A**) and G0PH97 (**B**) against LacNAc-ITag-1 (**4**). A peak of *m/z* 831 was observed for both, corresponding to galactosylated LacNAc-ITag-1. B4GALT4 demonstrated both UDP-Glc (**C**) and UDP-Gal (**D**) transfer onto Glc-ITag-1 (**1**). LgtH (**E**) demonstrated

transgalactosylation activity against GlcNAc-ITag-1 (**3**). A peak of *m/z* 669 was observed, corresponding to galactosylated GlcNAc-ITag-1 (LacNAc-ITag-1 **4**).

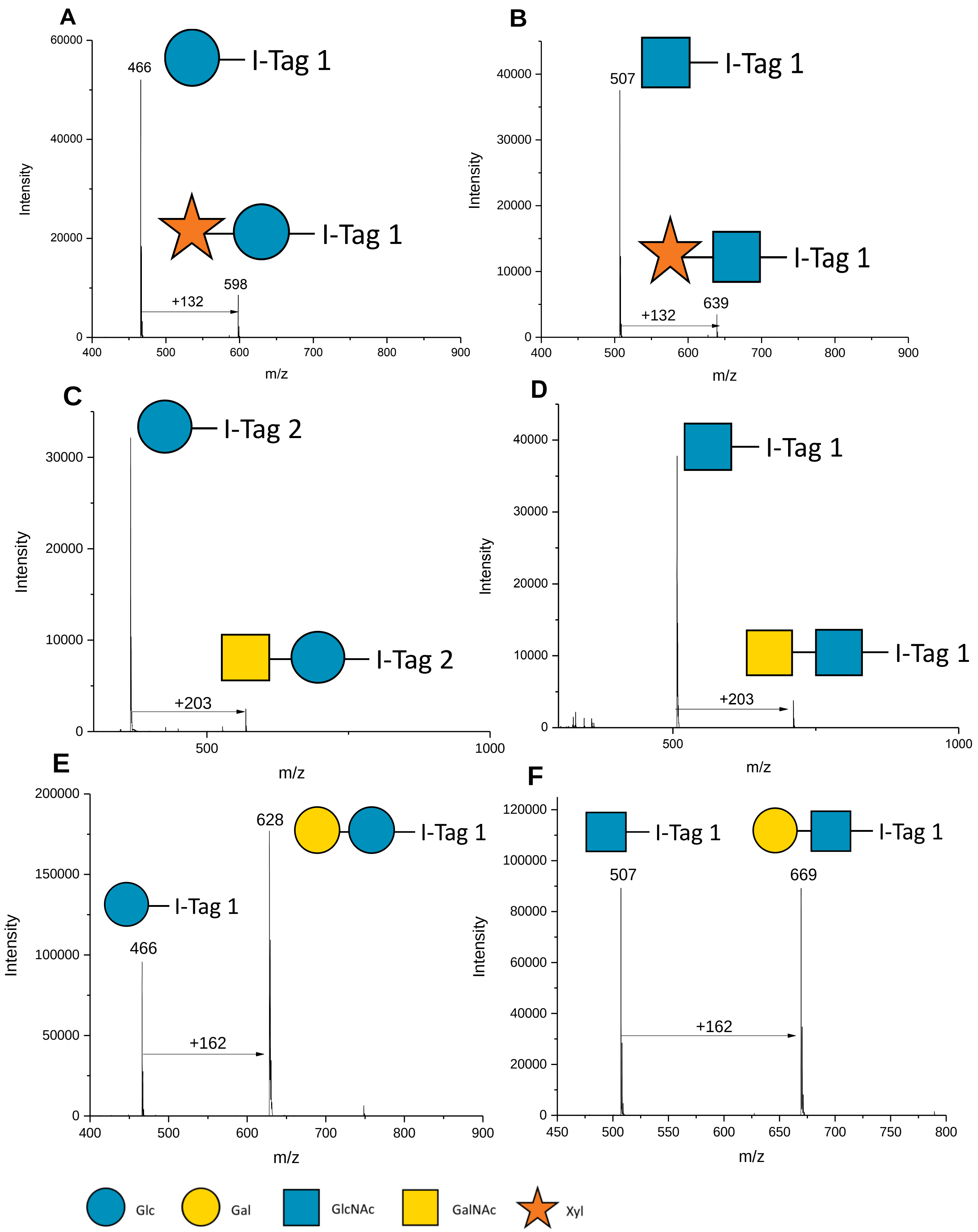

Supplementary figure 3 | Donor scope assessment of LgtB with ITagged acceptors
LgtB was able to transfer sugar from their respective UDP-conjugate when incubated with (**A** and **B**) UDP-Xyl; (**C** and **D**) UDP-GalNAc and (**E** and **F**) UDP-Gal onto ITag-Glc-1 (**1**) and ITag-GlcNAc-1 (**3**), respectively.

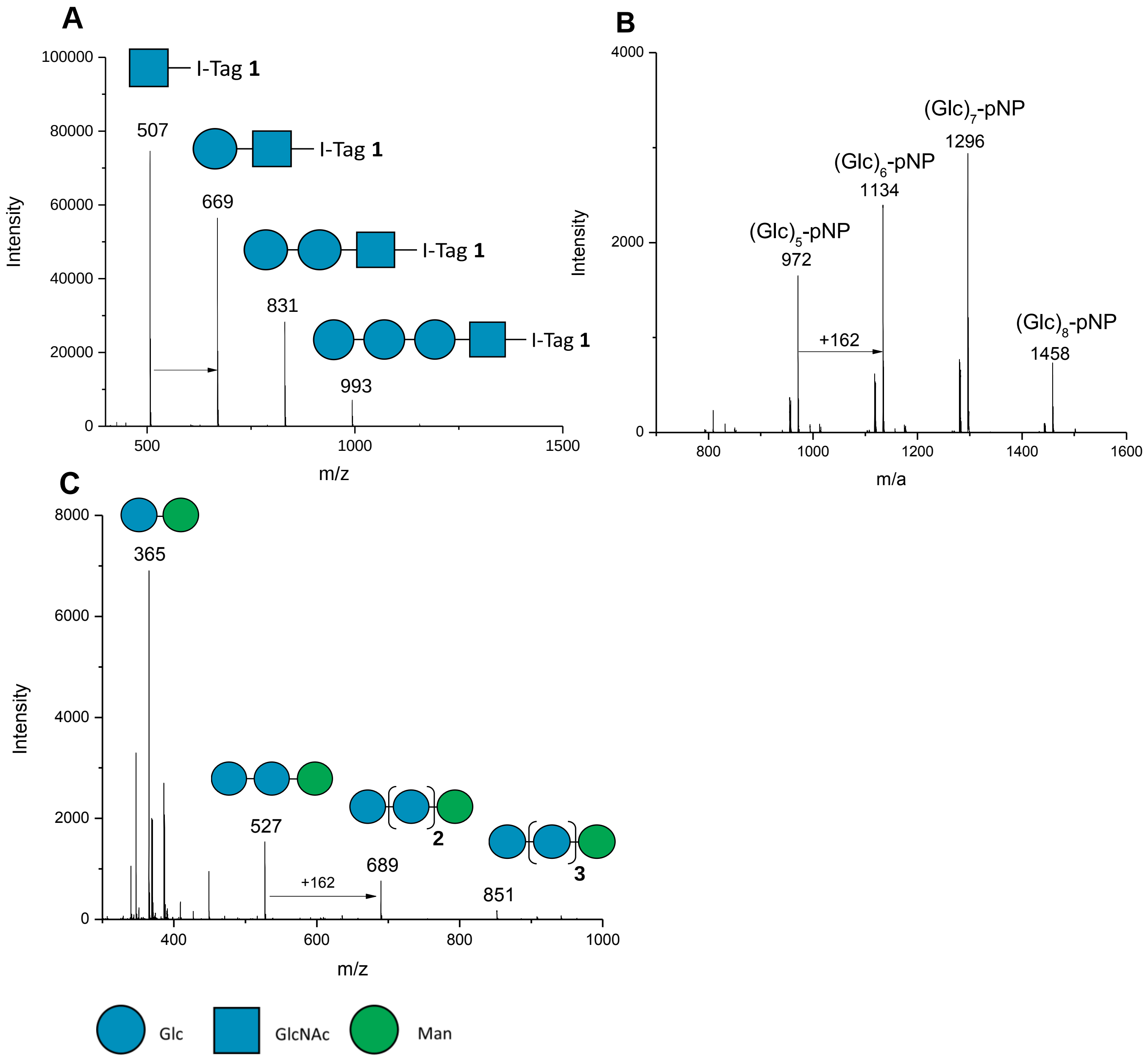

Supplementary figure 4 | Glucose polymerisation occurs onto a range of acceptors
Glc polymerisation by LgtB was observed via MALDI-TOF MS when using as acceptor substrates **A,** ITag-GlcNAc **3**, **B**, 4-Nitrophenyl β-D-glucose (Glc-pNP) and **C**, Mannose. Products are detected as [M] (for **A**) or $[M+Na^{+}]$ (for **B**, **C**)

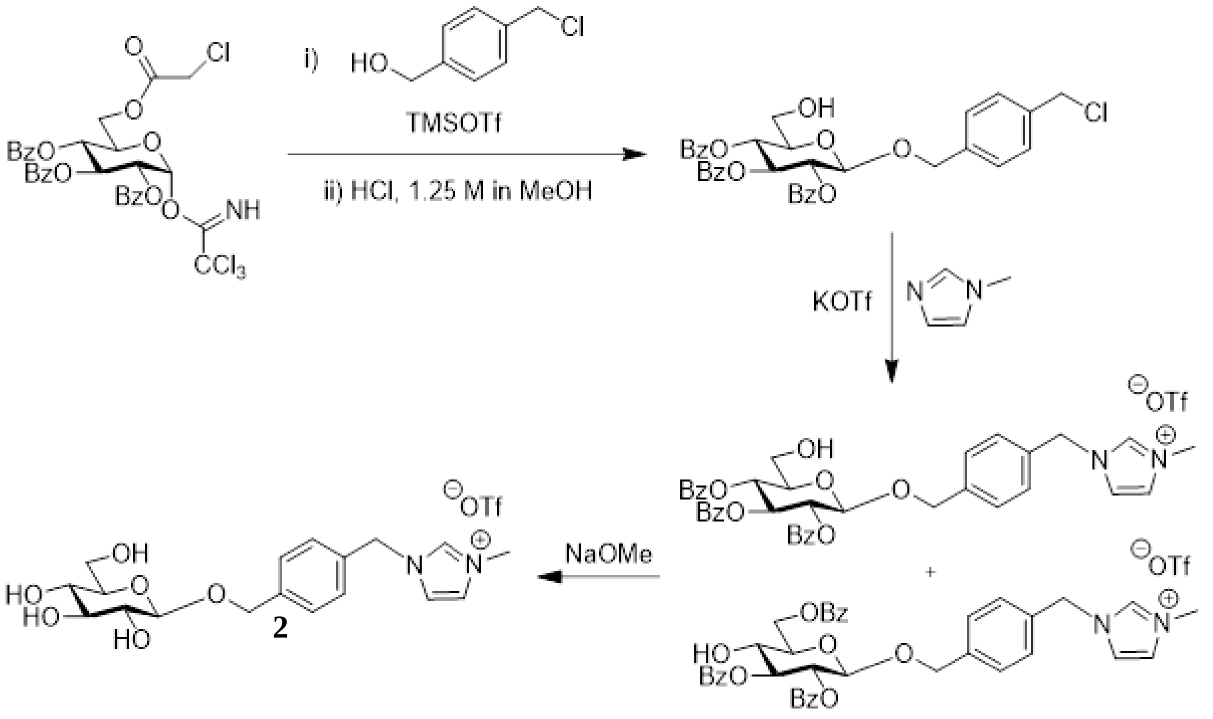

Supplementary figure 5 | Synthesis of 4-(1-Methyl-3-methyleneimidazolium)benzyl β-D-glucopyranoside trifluoromethanesulfonate (2)

Compound 2 was synthesised from 4-(chloromethyl)benzyl alcohol and glycosyl donor 2,3,4-tri-O-benzoyl-6-O-chloroacetyl-α-D-glucopyranosyl trichloroacetimidate in 4 steps with a yield of 49%, as described in detail in the methods section. Products were identified as follows: **IR** ν_{max}/cm^{-1} 3419br (OH), 3156w, 3113w, 2968w, 2929w, 1577, 1452, 1416, 1253s, 1225, 1160, 1076, 1028s, 758, 638, 574, 517; - 24˚ [*c* 1.08, MeOH]. **^{1}H NMR** δ_H (500 MHz, Methanol-*d*$_4$) 8.95 (1 H, s, NC*H*N), 7.58 (1 H, d, *J* 2.0, NC*H*CHN), 7.56 (1 H, d, *J* 2.0, NCHC*H*N), 7.53 – 7.48 (2 H, m, H_{arom}), 7.43 – 7.38 (2 H, m, H_{arom}), 5.39 (2 H, s, NCH_2), 4.94 (1 H, d, *J* 12.3, (C-1)OC*H*H), 4.70 (1 H, d, *J* 12.3, (C-1)OCH*H*), 4.35 (1 H, dd, *J* 7.7, 0.9, H-1), 3.92 (3 H, s, NCH_3), 3.89 (1 H, dd, *J* 12.0, 2.1, H-6a), 3.68 (1 H, dd, *J* 11.8, 5.5, H-6b), 3.37 – 3.23 (4 H, m, H-2, H-3, H-4, H-5); **^{13}C NMR** δ_C (126 MHz, Methanol-*d*$_4$) 140.64 (4˚ C_{arom} (CH_2O(C-1))) , 134.44 (4˚ C_{arom} (CH_2N)), 129.91, 129.65 (C_{arom}), 125.21 (NCH*C*HN), 123.60 (N*C*HCHN), 103.41 (C-1), 78.10, 78.04, 75.11, 71.66 (C-2, C-3, C-4, C-5), 71.07 ((C-1)O*C*H_2), 62.77 (C-6), 53.83 (N*C*H_2), 36.52 (N*C*H_3); **m/z** (ESI-HRMS) $C_{18}H_{25}N_2O_6^+$ ([M – OTf]$^+$) calculated: 365.1707; found 365.1712; (TLC-MS- (ESI)) $CF_3O_3S^-$ ([OTf]$^-$) calculated 149.0; found 148.8.

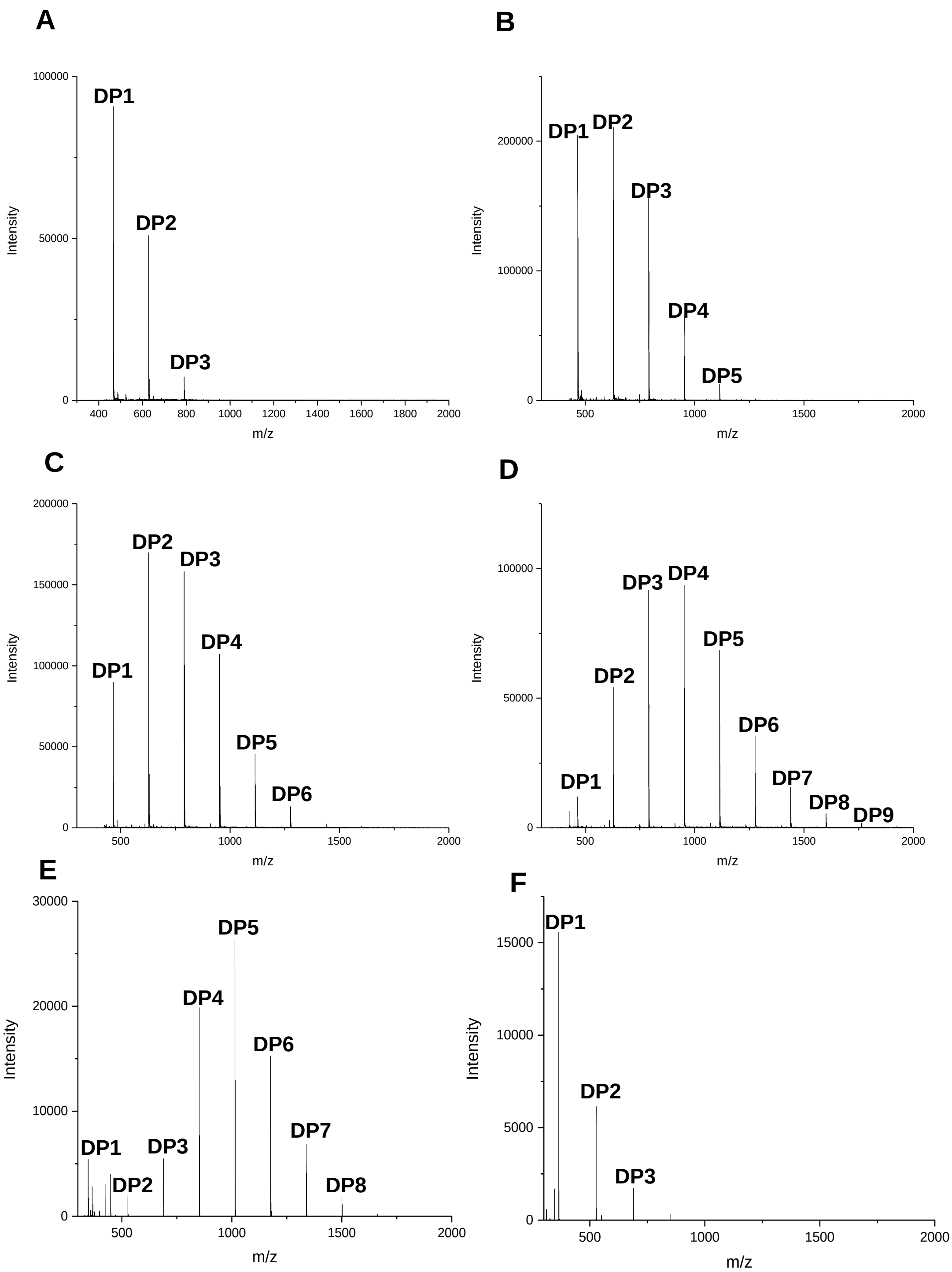

Supplementary figure 6 | Modification of reaction conditions drives oligosaccharide profile distribution

Product distribution broadens and degree of polymerisation increases during polymerisation of Glc onto Glc-ITag-1(**1**) after 96 h in response to LgtB concentration with **A** 0.17 mg ml^{-1} LgtB **B** 0.35 mg ml^{-1} LgtB, **C** 0.85 mg ml^{-1} LgtB, **D** 1.7 mg ml^{-1} LgtB. Starting concentration of acceptor affects oligosaccharide distribution, reactions analysed after 24 h with 1.7 mg ml^{-1} LgtB and **E** 0.2 mM Glc-ITag-2 (**2**) and **F** 0.5 mM **2**.

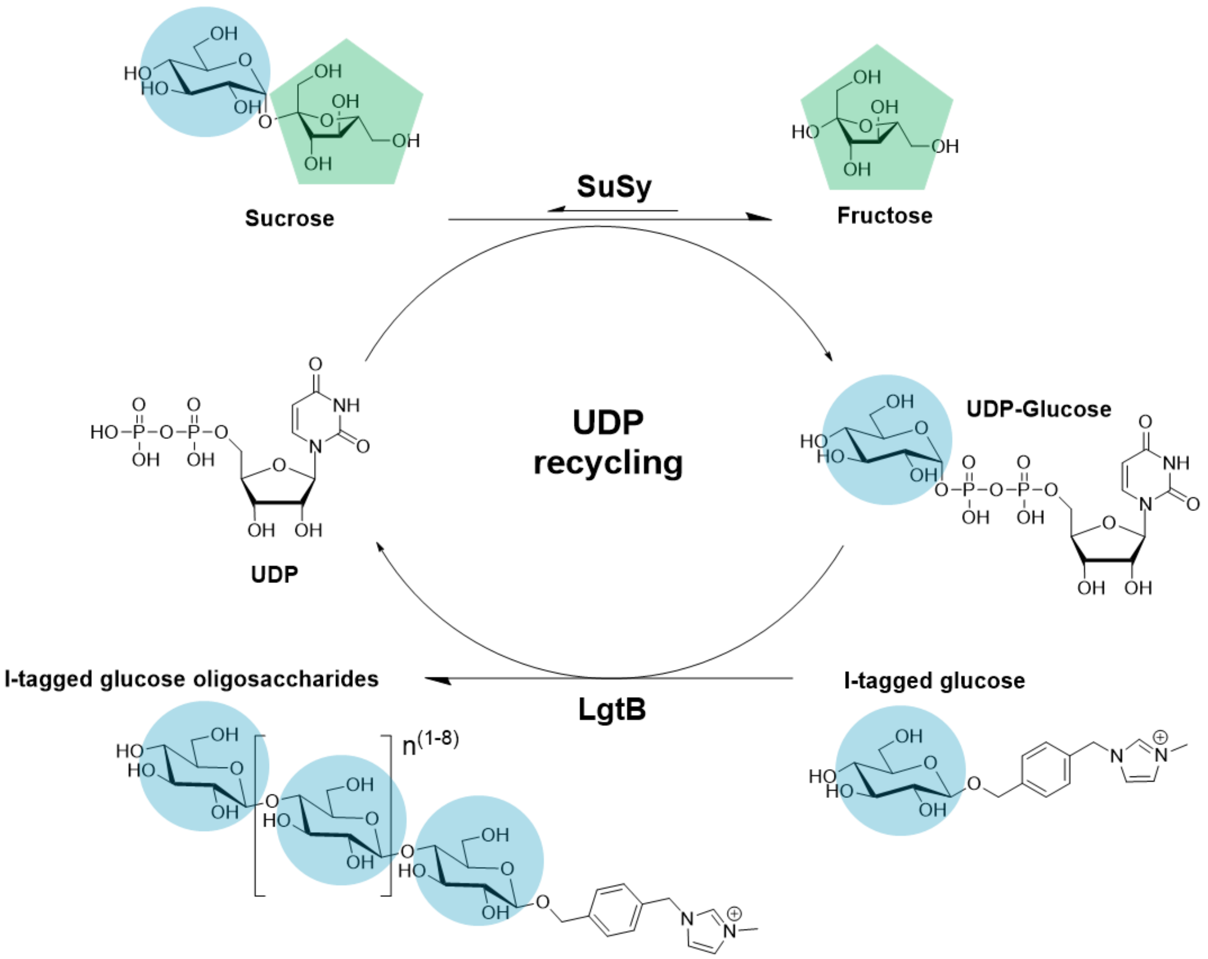

Supplementary figure 7 | Biocatalytic cascade enabling effective coupling of UDP-glucose regeneration with glucose polymerisation.

LgtB and sucrose synthase SuSy were utilised in a one-pot reaction for the production of ITagged cello-oligosaccharides.

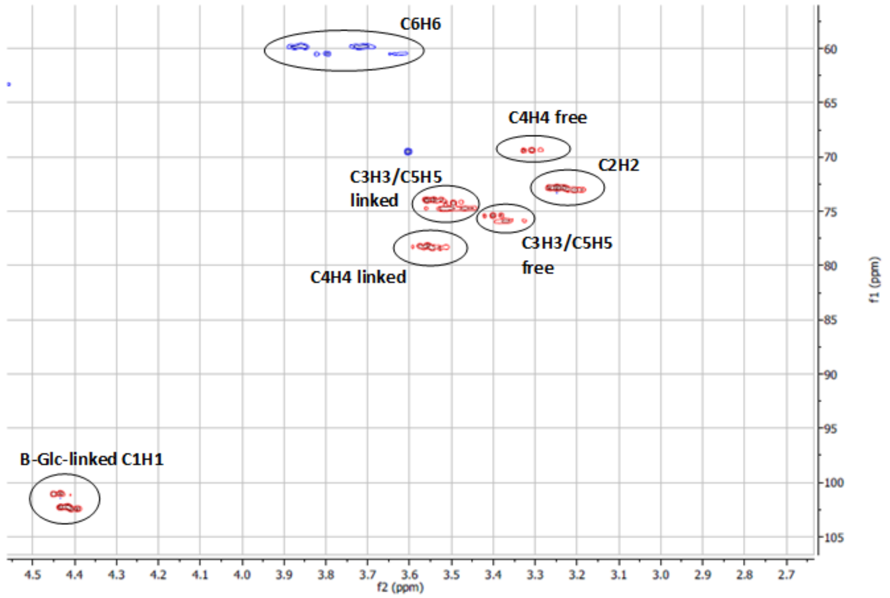

Supplementary figure 8 | HSQC confirmed that ITagged oligosaccharides generated by LgtB consist of β1,4-linked glucose

^{1}H-^{13}C gradient-selected sensitivity-enhanced multiplicity-edited HSQC of purified ITagged oligosaccharides generated by LgtB demonstrated the presence of β1,4-linked glucose.

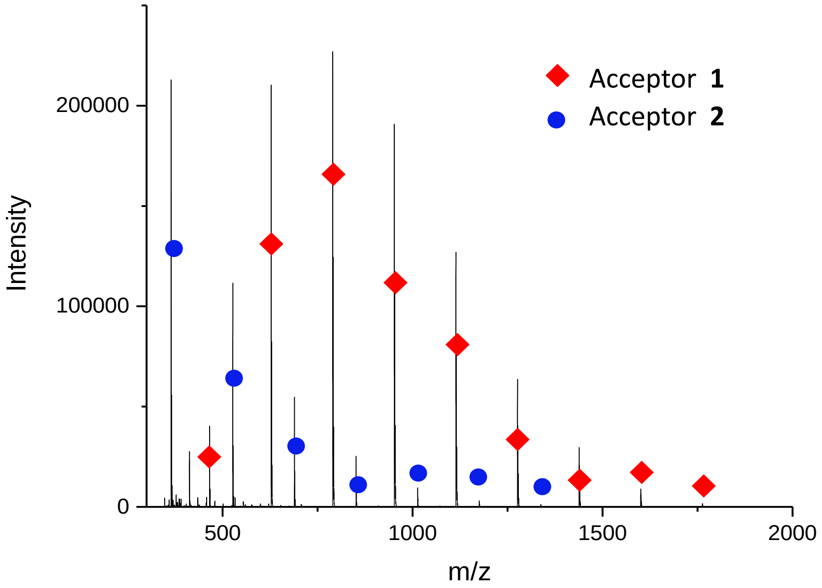

Supplementary figure 9 | SuSy cascade enables recycling of UDP for donor production

Superimposed MALDI-TOF spectra of oligosaccharide formation using the SuSy UDP recycling system, with Glc-ITag-1(**1**) and Glc-ITag-2 (**2**) as acceptors. Degree of polymerisation masses for **1** (red) as follows DP 1 (466), DP2 (628), DP3 (790), DP4 (952), DP5 (1114), DP6 (1276), DP7 (1438), DP8 (1600), DP9 (1762). Degree of polymerisation masses for **2** (blue) as follows DP 1 (365), DP2 (527), DP3 (689), DP4 (851), DP5 (1013), DP6 (1175), DP7 (1337).

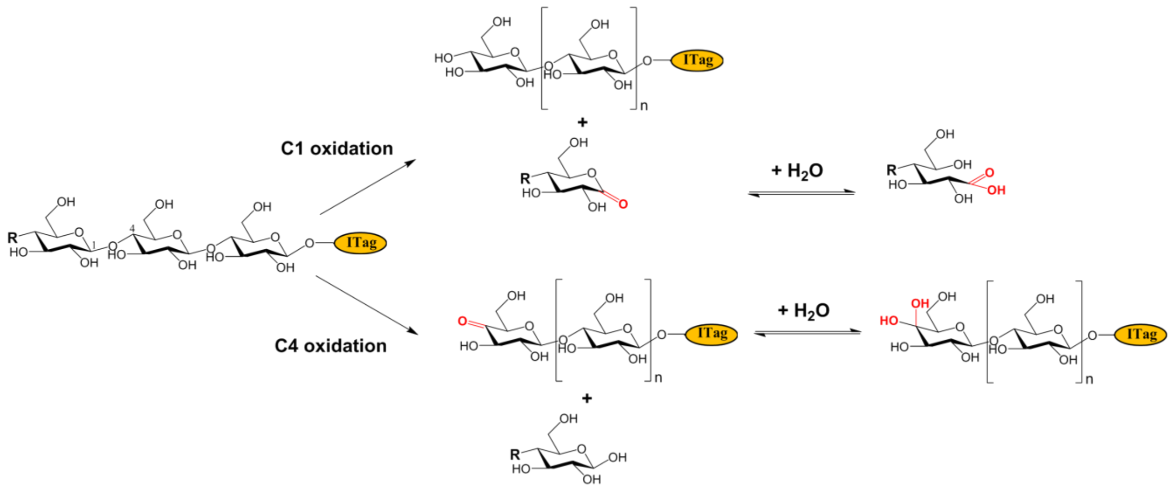

Supplementary figure 10 | LPMO reaction products
Scheme detailing the reaction products arising from C1- and C4 oxidation of ITagged cello-oligosaccharides

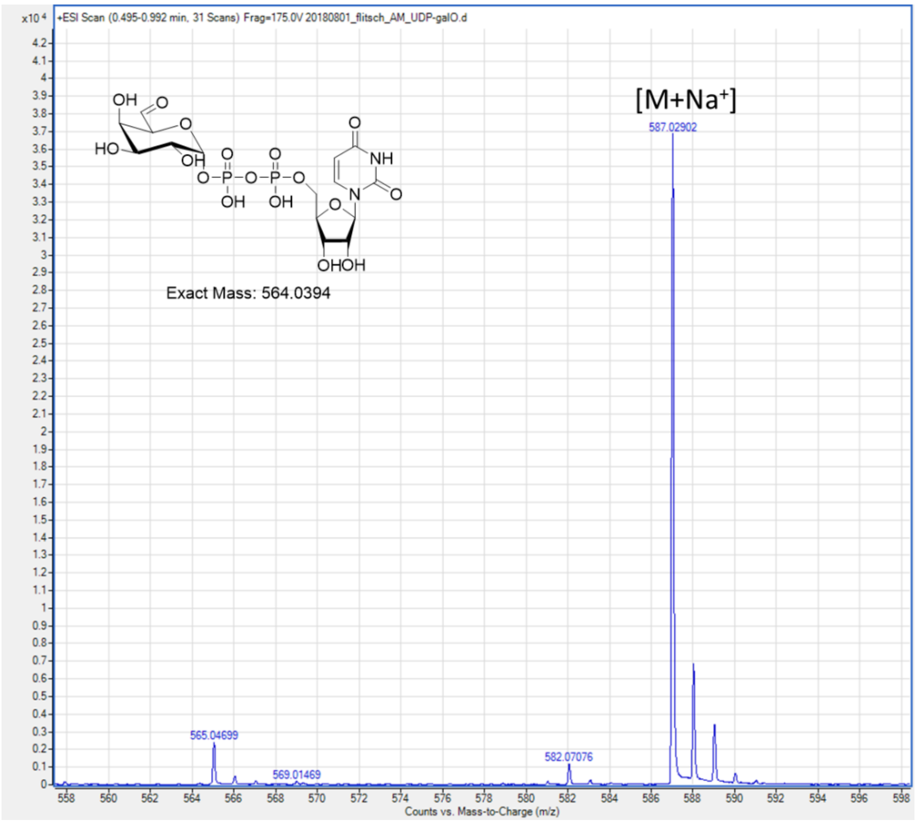

Supplementary figure 11 | Production of oxidised UDP-Gal
High-resolution mass spectrometry (HRMS) data confirming oxidised UDP-Gal has been produced via M_1 GOase.

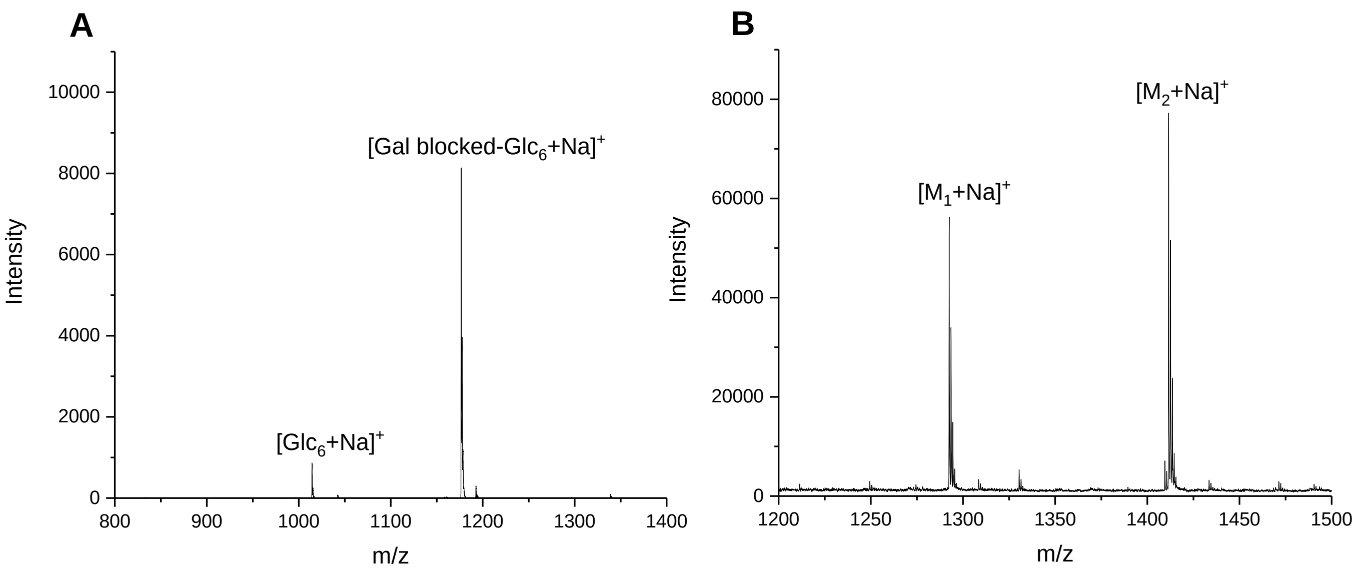

Supplementary figure 12| Galactosylation and hydrazide ligation of native cellooligosaccharide cellohexaose

A MALDI-TOF MS showing galactosylation of cellohexaose by LgtB (M = 1,175) with remaining cellohexaose substrate (M = 1,013). **B** MALDI-TOF MS showing sodiated adducts of single (M_1 = 1,270) and double (M_2 = 1,389) ligation of nicotinic hydrazide to the oxidised, galactose blocked cellohexaose. Double ligated species originate from nicotinic hydrazide ligating at the aldehyde of the Gal_{ox} as well as the aldehyde at the free reducing end. This procedure thus generated non-homogenous products with which it would prove difficult to assess the effectiveness of hydrolases.

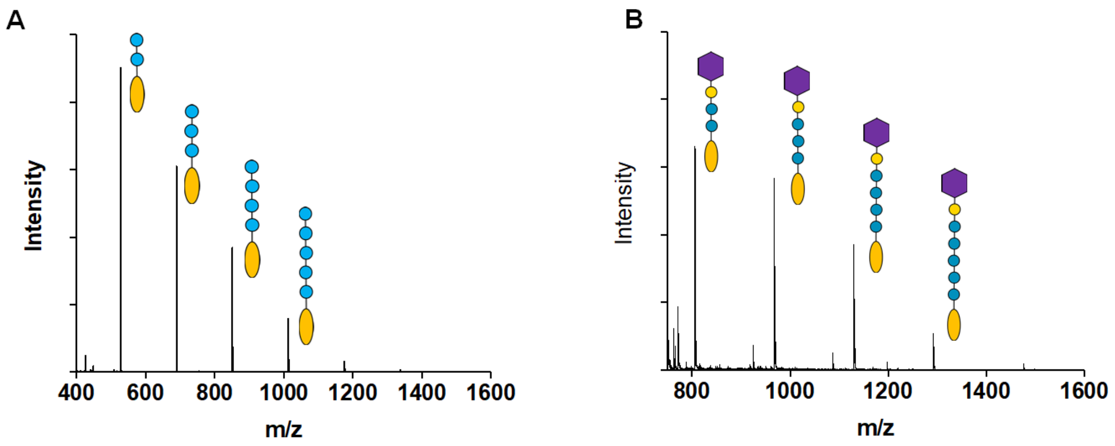

Supplementary figure 13| Galactosylation and hydrazide ligation of ITagged cello-oligosaccharides

A Glc-ITag-2 polymerised C18 purified envelope **B** Glc-ITag-2 polymerised C18 purified envelope following addition of oxidised Gal and subsequent nicotinic hydrazide ligation.

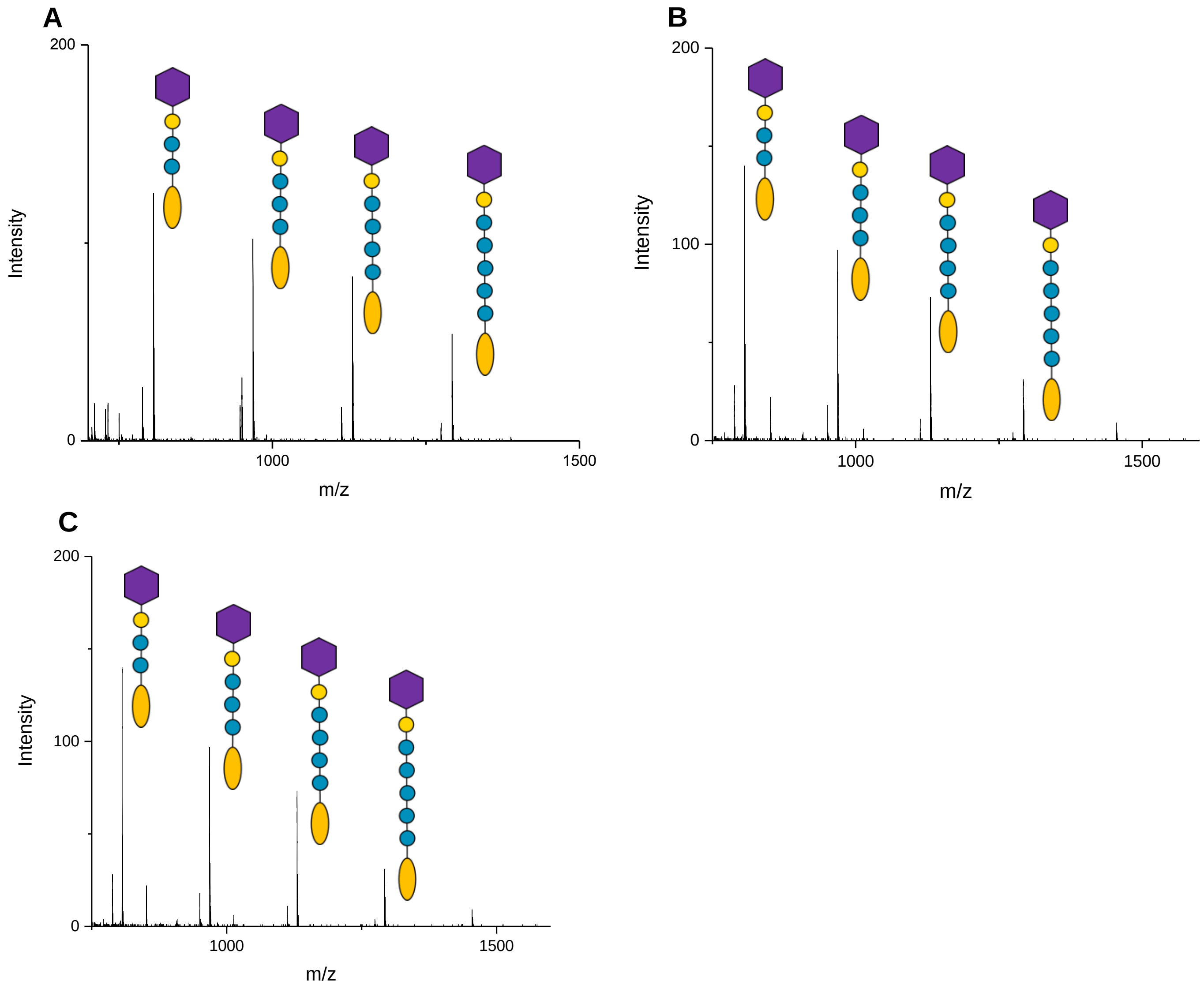

Supplementary figure 14| Confirmation of β-galactosidase activity blocking
MALDI-TOF MS showing nicotinic hydrazide ligated ITag-glucose oligosaccharides after incubation with β-galactosidases from glycoside hydrolase families **A** GH1 **B** GH42 and **C** GH50. Nicotinic hydrazide group represented by purple hexagon.

Supplementary Table 1 | LgtB activity on donor and acceptor substrates as detected by MALDI-TOF MS . (+) observed reaction products, (-) no observed reaction products.

Acceptors	Donors				
	UDP-Glc	UDP-Gal	UDP-Xyl	UDP-GalNAc	UDP-GlcNAc
Glc-ITag (**1**, **2**)	+	+	+	+	-
GlcNAc-ITag-1 (**3**)	+	+	+	+	-
Cellobiose (Glc-β1,4-Glc)	+	+		+	-
Glc	+	+			
GlcNAc	+	+			
GlcN	+	+			
β-GlcNAc-N3	+	+			
β-Glc-N3	+	+			
Glc-pNP	+	+			
Trehalose (Glc-α1,1-Glc)	-	-			
Man	+	+			
Sucrose (Glc-α1,2β-Fru)	+	+			
Cellotetraose-pNP	+	+			
Lactose (Gal-β1,4-Glc)	-	-			
Gal	-	-			
Xyl	-	-			
Ara	-	-			
UDP-Glc	-				

Materials and methods

Galactosyltransferase production

Galactosyltransferases LgtB (Uniprot: Q51116), LgtC (Uniprot: A0A3S5C3F9), LgtH (Uniprot: Q2TIJ3), B4GALT4 (Uniprot: B2RAZ5) and an uncharacterised homolog (Uniprot: G0PH97) were provided by Prozomix Lld, Haltwhistle, UK as purified protein suspended in $(NH_4)_2SO_4$. Samples were centrifuged (20000 g, 5 min, 4 °C), the supernatant was discarded and an equal volume of Tris buffer (20 mM pH 8.0) was added to resuspend the protein pellet. The protein sample was centrifuged again to remove precipitated protein and supernatant retained for analysis.

NMR of carbohydrates

NMR characterisation of ITagged glucose oligosaccharide linkages were performed using a ^{1}H-^{13}C gradient-selected sensitivity-enhanced multiplicity-edited HSQC on a Bruker AVIII 500 MHz spectrometer equipped with a QCI-F cryoprobe. Assignments were made based on the carbohydrate structure database (http://csdb.glycoscience.ru/database/).

Mass spectrometry of carbohydrates

Samples were prepared for analysis by MALDI-TOF via crystallisation with super DHB or THAP matrices. Super DHB (a 9:1 (w/w) mixture of 2,5-Dihydroxybenzoic acid and 2-hydroxy-5-methoxybenzoic acid) was prepared at 15 mg ml^{-1} in a mixture of 50 % (v/v) acetonitrile and 50 % (v/v) water containing 0.1 % trifluoroacetic acid (TFA). 2′,4′,6′-Trihydroxyacetophenone monohydrate (THAP) was prepared at 10 mg ml^{-1} in acetone. MALDI-TOF mass spectrometry was performed using the Bruker Ultraflex 3 in positive mode. MALDI-TOF was calibrated using peptide Calibration Standard II (Bruker) containing a range of peptides 757-3149 Da in size.

SuSy system for UDP recycling

Solanum lycopersicum Sucrose synthase (SLSUS6) was expressed in *E. coli* BL21(DE3) from a pET30 based vector containing an N-terminal Histag fusion as previously described[2]. Briefly, cells were cultured at 37 ºC, 250 rpm until reaching an OD600 of 0.6, after which cultures where moved to 18 ºC and protein expression was induced via addition of 1 mM IPTG for 20 h. Cells were harvested and lysed, after which cleared cell lysates were loaded onto an immobilised metal affinity chromatography (IMAC, GE Healthcare) Ni^{2+}-charged column. After washing 30 column volumes with 50 mM Tris, 50 mM NaCl, pH 8.0, SLSUS6 was eluted via addition of 500 mM imidazole. SuSy UDP-sugar regeneration reactions were carried out in a total volume of 50 µl containing 50 mM MES buffer (pH 6.0), 0.5 mM ITag substrate, 0.5 mM UDP, 35 mM sucrose, 5 mM $MgCl_2$, 2.8 µg SuSy and 20 µg LgtB. Reactions were incubated at 37 ^{o}C for 5 days and analysed by MALDI-TOF.

GalT screening panel

The panel of galactosyltransferases was screened in reactions as follows: 0.5 mM ITag acceptor (**1-4**), 1.5 mM UDP sugar donor, 10 mM $MgCl_2$, 10 mM $MnCl_2$, 0.1 mg ml^{-1} BSA, 50 mM Tris pH 8.0, and enzyme concentrations, one of the following: LgtH (0.3 mg ml^{-1}), G0PH97 (0.4 mg ml^{-1}), LgtC (0.1 mg ml^{-1}) and LgtB (1.7 mg ml^{-1}). Reactions were incubated at 37 ºC for 7 days to allow any potential polymerisation to occur. ITag acceptors Glc-ITag (**1**,**2**), GlcNAc-ITag-1 (**3**) and ITag-LacNAc-1 (**4**) were utilised in the screen along with UDP donors UDP-Glc and UDP-Gal.

Donor scope assessment and ITagged oligosaccharide production using LgtB

Final concentrations in ITag-glucose oligosaccharides experiments: 15 mM UDP-Glc, 1 mM ITag-glucose (**1**,**2**), 50 mM Tris HCl (pH 8.0), 10 mM $MgCl_2$, 10 mM $MnCl_2$, BSA 100 µg ml^{-1}, ~1.7 mg ml^{-1} LgtB (resuspended in dH_2O). Experiments assessing the activity of UDP-Xyl transfer onto ITag-glucose were as previous but with UDP-xylose 1mM final concentration, replacing UDP-glucose as the donor. ITag-glucose oligosaccharides were purified on a 5 ml C18 column using a gradient of methanol in water with concentrations ranging from 0 to 100% (v/v). All fractions were collected and analysed via MALDI-TOF MS, and those containing purified oligosaccharides were pooled.

Enzymatic digestions of ITagged-oligosaccharides

β-glucosidase from almond (49290, Sigma-Aldrich) was dissolved in water at 1 mg ml^{-1}. Cellulase from *Aspergillus niger* (C1184**,** Sigma-Aldrich) was dissolved in pH 5.0, sodium acetate buffer at 10 mg ml^{-1}. Reactions were performed at pH 5.0, 37 °C for 2 h at concentrations of 0.5 mg ml^{-1} and 5 mg ml^{-1} β-glucosidase and cellulase respectively with 10 % (v/v) C18 column purified ITagged glucose oligosaccharides. LPMO reactions were set up as follows: 100 µM H_2O_2, 200 µM ascorbate and 5 µM LPMO in 25 mM Tris-HCl buffer pH 7.5 and performed at room temperature for 14 h.

LPMO production

The synthesised genes coding for *Neurospora crassa* NcLPMO9C (Uniprot: Q7SHI8) and *Thermobifida fusca Tf(*AA10)B (Uniprot: Q47PB9) were cloned into pET22b vector (encoding a pelB sequence for periplasmic secretion) and expressed using *E. coli* C43(DE3), via periplasmic secretion to obtain an N-terminal His. After growth at 37°C until OD600 of 0.6, protein expression was induced with 0.1mM IPTG at 25°C for 16 h, 200 rpm. *Lentinus similis* LsLPMO9A (Uniprot: A0A0S2GKZ1) was expressed as an E8K-vector construct in *E. coli DH5a* at 25 °C for 24 h, 200 rpm. Protein production was induced using 10 mM arabinose.

The cells were harvested by centrifugation (4000 rpm, 20 min, 4°C) and lysed in equilibration buffer (50 mM sodium phosphate (NaPi) buffer pH 8.0, containing 300 mM NaCl, 1 mg ml^{-1} lysozyme and 10 µg ml^{-1} DNase,) via sonication (10 min, 1s on 1s off, 40 % sonotrode power). Cell debris was removed using centrifugation at 20000 rpm 30 min at 4°C and cleared supernatant was applied to a pre-equilibrated 5 ml Strep-tactin superflow cartridge (Qiagen). The column was washed with equilibration buffer and the protein eluted using 5mM desthiobiotin. Fractions containing pure target protein, identified via SDS-PAGE, were pooled, concentrated and re-buffered into 25 mM Tris-HCl pH 7.5 using PD10 desalting columns. The protein was loaded with 5x molar excess $CuCl_2$ for 1 h at 4 °C and the excess Copper was then removed using PD10 desalting columns. The protein was stored at -80 °C, 25 mM Tris-HCl buffer pH 7.5. Activity of purified enzymes was confirmed via detection

of the LPMO side reaction that reduces O_2 to H_2O_2 in the absence of substrate, via a peroxidase-coupled assay as described previously[3].

Endo-substrate development

M_1 Galactose oxidase (GOase) was expressed and purified as previously described[4]. Oxidised UDP-Gal (UDP-Gal_{Ox}) was produced as follows: 0.1 mg ml^{-1} horseradish peroxidase (HRP), 1 mg ml^{-1} GOase M_1, 0.1 mg ml^{-1} catalase, 10 mM UDP-galactose, NaPi pH 7.4 reaction was incubated until completion at 25 ^oC, 250 rpm for 4 h filtered on a viva spin column to remove protein, desalted on a Sephadex G-10 column then freeze dried. Final concentrations in oxidised UDP-Gal blocking of cellohexaose were as follows: 10 mM UDP-Gal_{Ox}, 5 mM cellohexaose, 10 mM $MnCl_2$, 50 mM NaPi (pH 8), LgtB (1.7 mg ml^{-1}). Final concentrations in Gal_{Ox} blocking of ITagged glucose oligosaccharides were as follows: 10 mM UDP-Gal_{Ox}, 20 % (v/v) C18 column purified ITagged glucose oligosaccharides, 10 mM $MnCl_2$, 50 mM sodium phosphate (pH 8), LgtB (1.7 mg ml^{-1}). After incubation at 250 rpm, 37 $^{\circ}C$ for 16 h, reaction products were purified on a viva spin column (10000 MWCO). Subsequent hydrazide ligation required 20 equivalents of nicotinic hydrazide addition to the previous reaction mixes, which were then incubated at 30 $^{\circ}C$, 250 rpm for 2 h.

Enzymatic galactosylation of native cello-oligosaccharides (Fig. S11a) followed by oxidation of the terminal Gal then nicotinic hydrazide ligation (Fig. S11b) was also demonstrated. Final concentrations in galactose capping of cellohexaose were as follows: 10 mM UDP-Gal, 5 mM cellohexaose, 10 mM $MnCl_2$, 50 mM NaPi (pH8), LgtB (1.7 mg ml^{-1}). Reaction was incubated at 250 rpm, 37 $^{\circ}C$ for 16 h. Reaction was then purified on a viva spin column. Product was then oxidised, final concentration during the oxidation of the galactose capped cellohexaose were as follows: 0.1 mg ml^{-1} HRP, 1 mg ml^{-1} GOase M_1, 0.1 mg ml^{-1} catalase, 0.5 mM galactosylated cellohexaose, NaPi pH 7.4. Reaction was incubated at 25 $^{\circ}C$, 250 rpm for 4 h then treated with nicotinic hydrazide as in previous method.

Nicotinic hydrazide ligated ITagged cello-oligosaccharides were incubated with β-galactosidases to ensure inaccessibility of exo-active cellulolytic enzymes. β-Galactosidase 1A from Sulfolobus solfataricus (CZ04141), β-Galactosidase 42A from *Caldicellulosiruptor saccharolyticus* (CZ09741) and β-Galactosidase 50A from *Victivallis vadensis* (CZ05161) were purchased from NZYtech. Reactions with GH1 were incubated at 90 $^{\circ}C$ for 2 h in reactions containing: nicotinic hydrazide ligated ITagged cello-oligosaccharides (25 % v/v), 50 mM pH 5 sodium acetate buffer, 0.5 mg ml^{-1} GH1. Reactions with GH42 were incubated at 65 $^{\circ}C$ for 2 h, in reactions containing: nicotinic hydrazide ligated ITagged cello-oligosaccharides (25 % v/v), 50 mM pH 4 sodium acetate buffer, 0.5 mg ml^{-1} GH42. Reactions with GH50 were incubated at 40 $^{\circ}C$ for 2 h, in reactions containing nicotinic hydrazide ligated ITagged cello-oligosaccharides (25 % v/v), 50 mM pH 7 Tris buffer, 0.25 mg ml^{-1} GH50.

Synthesis of 4-(1-Methyl-3-methyleneimidazolium)benzyl β-D-glucopyranoside trifluoromethanesulfonate (2) (Glc-ITag-2)

Glycosyl acceptor 4-(chloromethyl)benzyl alcohol (0.0689 g, 0.440 mmol, 1 eq) and glycosyl donor 2,3,4-tri-O-benzoyl-6-O-chloroacetyl-α-D-glucopyranosyl trichloroacetimidate (0.6277 g, 0.880 mmol, 2.0 eq) were placed in a dry vial and dried under vacuum for 30 min. 2.20 ml of anhydrous DCM was added to the donor/acceptor vial under nitrogen, resulting in a solution of volume 2.75 ml and therefore approximately 0.160 M in acceptor and 0.320 M in donor. A stock solution of TMSOTf (0.06 M in DCM) was made by dissolving TMSOTf (0.217 ml, 1.200 mmol) in 20 ml anhydrous DCM. 3.0 ml of this stock solution was used for this reaction. The flow microreactor and attached tubing was flushed with nitrogen. The donor/acceptor solution and TMSOTf solution were each taken up in a syringe and installed onto a syringe pump. The solutions were then injected into the microreactor (total internal volume of reactor chip and outlet tubing = 32.8 μL) at the desired flow rate corresponding to the residence time (15 seconds, 65.60 μl min^{-1} in each syringe for a combined flow rate of 131.20 μl min^{-1} in reactor zone) via the inlet tubing. The flow reaction was performed at RT. The mixture that flowed from the microreactor was dropped in a flask containing reagent grade DCM in air to quench the reaction. Reaction solution was collected for 41 min 30 sec, after which time the reaction mixture solvent was removed under reduced pressure. The crude product was dissolved in DCM (20 ml) and washed with water (8 ml), then the water was extracted with a further portion of DCM (20 ml). The DCM fractions were collected, dried with magnesium sulfate, filtered and the solvent was removed under reduced pressure. The dried residue was dissolved in a minimal volume of DCM, then HCl, 1.25 M in MeOH (7.04 ml, 8.800 mmol) was added. The resulting solution was stirred for 16 h at RT in air, then diluted with DCM (15 ml) and water (10 ml) and product was extracted into the DCM phase. The aqueous phase was washed with a further DCM portion (15 ml) then DCM washings were combined, dried using magnesium sulfate, filtered and the solvent was removed under reduced pressure. The dried residue was washed with hexane (3 x 5 ml), then dried under reduced pressure for 1 h, before being dissolved in anhydrous MeCN (5 ml) under a nitrogen atmosphere. 1-Methyl imidazole (0.14 ml, 1.76 mmol) and potassium trifluoromethanesulfonate (0.3312 g, 1.76 mmol) were added and the resulting mixture was heated under reflux at 90 ˚C and stirred for 18 h, after which time TLC (DCM:MeOH 94:6) showed the reaction to be complete by MS. Solvent was removed under reduced pressure, then DCM (5 ml) and 1 M HCl $_{aq.}$ (5 ml) were added to the residue and product was extracted into the DCM phase. The aqueous phase was washed with DCM (2 x 5 ml), then the DCM portions were combined, dried using magnesium sulfate, filtered and the solvent was removed under reduced pressure. The crude residue was washed with neat Et_2O (5 ml) and DCM:Et_2O 5:95 (2 x 5 ml). The ITagged products were dissolved in methanol (2.2 ml) and sodium methoxide (50.0 μl, 0.22 mmol, 25 % wt in MeOH) was

added. The solution was stirred at RT for 3 h after which time TLC-MS showed the reaction to be complete. The solution was then brought to pH 7 using 1 M HCl $_{aq.}$. Solvent was removed, then the residue was diluted with DCM and water and product was extracted into the aqueous phase. Water was removed under reduced pressure and the dried mixture was purified by reversed-phase HPLC (Water:MeCN) to yield the **ITag-glucose 2 (2)** (0.1110 g, 49 % over 4 steps) as a solid.

^{1}**H NMR** δ_{H} (500 MHz, Methanol-d_4) for ITag-glucose **2**

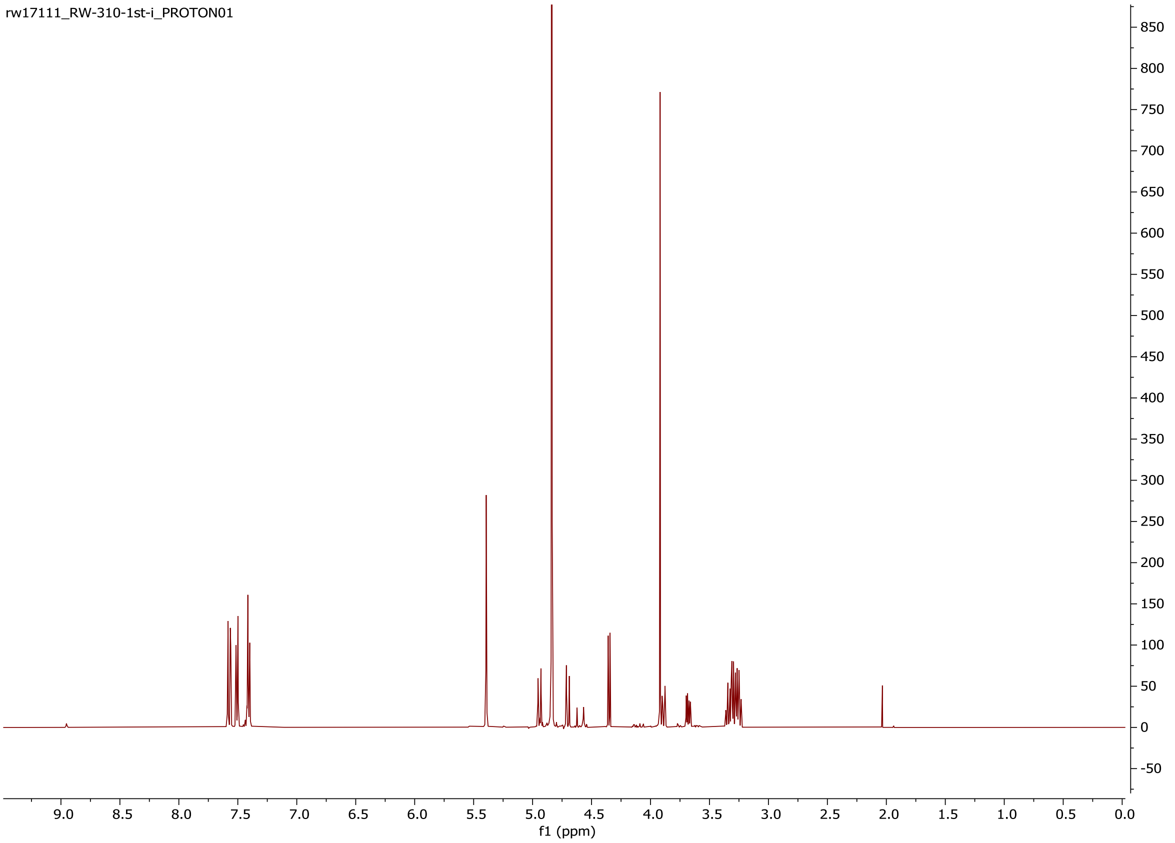

^{13}C NMR δ_{C} (126 MHz, Methanol-*d_4*) for ITag-glucose **2**

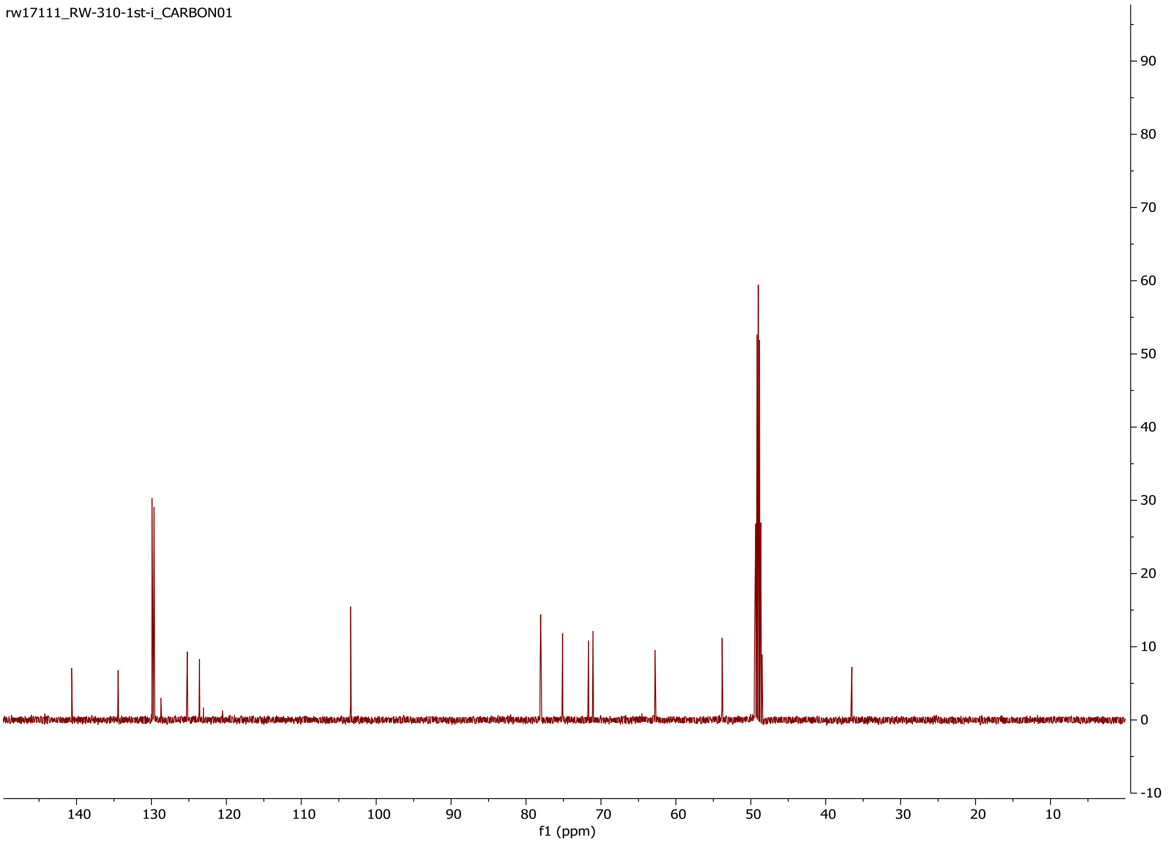

References

(1) Huang, K.; Parmeggiani, F.; Ledru, H.; Hollingsworth, K.; Mas Pons, J.; Marchesi, A.; Both, P.; Mattey, A. P.; Pallister, E.; Bulmer, G. S.; et al. Enzymatic Synthesis of N-Acetyllactosamine from Lactose Enabled by Recombinant Β1,4-Galactosyltransferases. *Org. Biomol. Chem.* **2019**, *17* (24), 5920–5924.

(2) Duan, X. C.; Chen, H.; Liu, F. F.; Conway, L.; Wei, S.; Cai, Z. P.; Liu, L.; Voglmeir, J. One Assay for All: Exploring Small Molecule Phosphorylation Using Amylose-Polyiodide Complexes. *Anal. Chem.* **2015**, *87* (19), 9546–9550.

(3) Kittl, R.; Kracher, D.; Burgstaller, D.; Haltrich, D.; Ludwig, R. Production of Four Neurospora Crassa Lytic Polysaccharide Monooxygenases in Pichia Pastoris Monitored by a Fluorimetric Assay. *Biotechnol. Biofuels* **2012**, *5* (1), 79.

(4) Herter, S.; McKenna, S. M.; Frazer, A. R.; Leimkühler, S.; Carnell, A. J.; Turner, N. J. Galactose Oxidase Variants for the Oxidation of Amino Alcohols in Enzyme Cascade Synthesis. *ChemCatChem* **2015**, *7* (15), 2313–2317.
